# Exploring the Multifaceted Landscape of MASLD: A Comprehensive Synthesis of Recent Studies, from Pathophysiology to Organoids and Beyond

**DOI:** 10.3390/biomedicines12020397

**Published:** 2024-02-08

**Authors:** Allison Soto, Colby Spongberg, Alessandro Martinino, Francesco Giovinazzo

**Affiliations:** 1 Department of Surgery, University of Illinois College of Medicine, Chicago, IL 60607, USA; asoto59@uic.edu; 2 Touro College of Osteopathic Medicine, Great Falls, MT 59405, USA; 3 Department of Surgery, Duke University, Durham, NC 27708, USA; 4 General Surgery and Liver Transplant Unit, Fondazione Policlinico Universitario Agostino Gemelli IRCCS, 00168 Rome, Italy

**Keywords:** MASLD, NAFLD, organoids, pathophysiology

## Abstract

Non-alcoholic fatty liver disease (NAFLD) is a widespread contributor to chronic liver disease globally. A recent consensus on renaming liver disease was established, and metabolic dysfunction-associated steatotic liver disease, MASLD, was chosen as the replacement for NAFLD. The disease’s range extends from the less severe MASLD, previously known as non-alcoholic fatty liver (NAFL), to the more intense metabolic dysfunction-associated steatohepatitis (MASH), previously known as non-alcoholic steatohepatitis (NASH), characterized by inflammation and apoptosis. This research project endeavors to comprehensively synthesize the most recent studies on MASLD, encompassing a wide spectrum of topics such as pathophysiology, risk factors, dietary influences, lifestyle management, genetics, epigenetics, therapeutic approaches, and the prospective trajectory of MASLD, particularly exploring its connection with organoids.

## 1. Introduction

Non-alcoholic fatty liver disease (NAFLD) is a widespread contributor to chronic liver disease globally. Its disease spectrum involves hepatic steatosis in the absence of alternative causes like excessive alcohol consumption. The range extends from the less severe non-alcoholic fatty liver (NAFL) to the more intense non-alcoholic steatohepatitis (NASH), characterized by inflammation and apoptosis. NAFLD has the potential to advance to fibrosis and cirrhosis, with NAFL presenting hepatic steatosis without inflammation, while NASH involves both inflammation and apoptosis, and possibly leads to fibrosis and cirrhosis [1]. 

The term NAFLD (non-alcoholic fatty liver disease) is currently undergoing a shift towards adopting a more precise representation of the heterogeneity of the disease, all while being cognizant of addressing associated stigmas [2]. A consensus on renaming liver diseases was established via a modified Delphi method, with input from 236 experts across 56 countries, coordinated by three leading liver associations, and requiring a 67% supermajority for agreement. The adoption of “Steatotic liver disease” as a unifying term resulted from the majority opinion (between 61% and 66%) that the terms “non-alcoholic” and “fatty” were pejorative, while maintaining “steatohepatitis” for its significant pathophysiological implications. As such, the consensus agreed that metabolic dysfunction-associated steatotic liver disease, MASLD, should be the preferred terminology. Furthermore, a diagnosis of MASLD now requires the presence of at least one out of five cardiometabolic risk factors. In concordance with MASLD, metabolic dysfunction-associated steatohepatitis (MASH) is now the replacement for NASH. Cases without metabolic indicators and identifiable causes are now classified under cryptogenic steatotic liver disease. Additionally, a novel category named Metabolic and Alcohol Related/Associated Liver Disease (MetALD) has been established for MASLD patients with higher alcohol consumption (140–350 g/week for women and 210–420 g/week for men). This updated classification and diagnostic approach is broadly endorsed for its non-stigmatizing nature, and it contributes to improved awareness and the more precise identification of patients [3]. In this review, we will be adhering to the new convention and addressing this disease process as MASLD.

According to the US guidelines for MASLD management, MASLD is defined as the presence of steatosis with at least 5% of fat infiltration in imaging or histology, and it excludes cases of steatosis caused by alcohol, drugs, or viruses that induce steatosis [1]. A liver biopsy maintains its status as the gold standard for diagnosing, while ultrasound is recommended as the primary screening tool for steatosis in specific populations. The identification of advanced fibrosis among individuals with MASLD is achieved by reliably excluding advanced fibrosis using tools such as the MASLD fibrosis score, FIB-4 score, or transient elastography. Although advanced technologies like Magnetic Resonance can precisely assess steatosis and determine the stage of fibrosis, their routine use is not yet standardized [4].

The worldwide prevalence of MASLD is 30% and is rising rapidly according to a systematic review and meta-analysis collected from 1990 to 2019, with the highest MASLD prevalence in Latin America at 44.37% [5]. Individuals with MASLD commonly display features of metabolic syndrome (MS), including systemic hypertension, dyslipidemia, insulin resistance, or overt diabetes; furthermore, 50 to 70% of individuals with diabetes have MASLD [6]. Visceral obesity has been identified as a risk factor for MASLD, and it is crucial to note that MS is a recognized contributor to the development of cardiovascular disease. While cardiac and vascular diseases are observed as primary causes of mortality in MASLD patients, the exact pathophysiological mechanisms linking cardiovascular disease and MASLD are still not fully elucidated. Research has depicted that insulin resistance is believed to be a shared factor in the pathogenesis connecting the conditions of MASLD and cardiovascular disease [1]. MASLD is the fastest growing medical condition leading to hepatocellular carcinoma, with patients subsequently needing liver transplantations [7].

This research project endeavors to comprehensively synthesize the most recent studies on MASLD. As organized in Table 1, this paper encompasses a wide spectrum of topics such as pathophysiology, risk factors, dietary influences, lifestyle management, genetics, epigenetics, therapeutic approaches, and the prospective trajectory of MASLD, particularly exploring its connection with organoids.

## 2. Pathophysiology

The direct development of MASLD is not completely understood, but a complex set of processes is thought to lead to the underlying fatty infiltration of the liver. The reasons for this fatty liver infiltration have been assumed to be encompassed under an umbrella term that includes all causes (mostly DM2 or obesity) and are other than alcohol. Although the true pathogenesis of MASLD is unknown, the pathogenesis according to the most current literature is thought to occur due to a “first hit” and subsequent “second hit”. The “first hit” is believed to be from acquired insulin resistance and hepatic steatosis caused by excess fatty acids [1]. This excess of fatty acids and the accumulation of lipid droplets in hepatocytes leading to this “first hit” are heightened by genetic variants, and, unlike free fatty acids or triglycerides, free cholesterol is found to sensitize the liver to steatohepatitis [8]. Next, a “second hit” is thought to occur due to oxidative stress, lipid peroxidation, inflammation, and fibrosis, leading to pathological changes in hepatocytes. MASLD can lead to cirrhosis and hepatocellular carcinoma, and, therefore, it is an important factor in mortality resulting from liver-related diseases [1].

**Figure 1:**
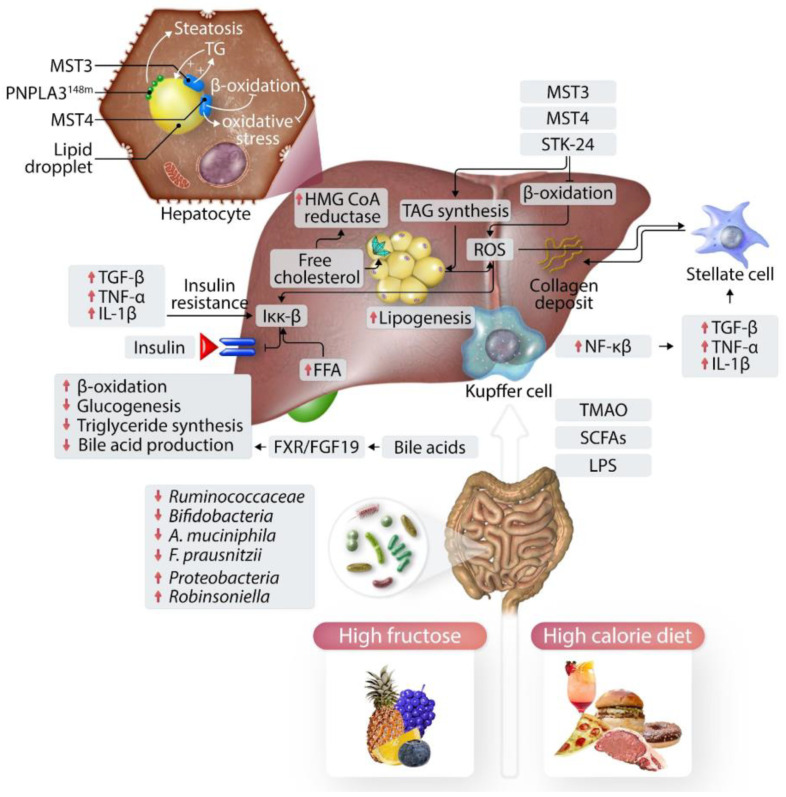
The onset and progression of MASLD are influenced by a confluence of dietary habits and the resulting changes in the intestinal flora, which in turn affect liver function and fat deposition. The diagram depicts a series of interconnected biochemical and cellular responses: an increase in free fatty acids (FFAs), elevated production of reactive oxygen species (ROS), and persistent minor inflammation disrupt the normal metabolic function by influencing IKK-β, leading to insulin resistance. The upsurge in the body’s fat creation and accumulation of free cholesterol within the hepatocytes intensifies cellular distress, also referred to as lipotoxicity. The presence of lipopolysaccharides (LPS) prompts liver Kupffer cells to secrete inflammatory cytokines. The byproducts such as short-chain fatty acids (SCFAs) and trimethylamine-N-oxide (TMAO) are generated by gut bacteria from dietary intake. These elements synergistically spark inflammation, such as by the generation of inflammatory cytokines, which consequently trigger stellate cells in the liver to produce collagen, leading to the development of fibrosis. In the figure, the arrows indicate changes in the level of activity or concentration of specific factors: upward arrows point to an increase, such as a rise in TGF-β, TNF-α, and IL-1β, which are indicators of inflammation and fibrogenic activity. Downward arrows signify a decrease, as observed with β-oxidation, gluconeogenesis, triglyceride synthesis, and bile acid production, which are processes typically downregulated in NAFLD. This visual representation serves to illustrate the dynamic and multifaceted pathophysiological mechanisms at play in MASLD [8].

MASLD can be classified into two distinct types. The first type exhibits a close association with metabolic syndrome, and current theories posit insulin resistance as the primary underlying pathophysiological mechanism. On the other hand, the second type of MASLD is linked to infectious pathologies, which can contribute to the development of liver steatosis. In this scenario, infections such as hepatitis C and HIV may play a role, along with medications (such as total parenteral nutrition, glucocorticoids, tamoxifen, tetracycline, amiodarone, methotrexate, valproic acid, and vinyl chloride), specific toxins (including alcohol), or inherited/acquired metabolic disorders (such as lipodystrophy, cachexia, or intestinal bypass surgery) [1]. As shown in Figure 1, the pathogenesis of MASLD is multifactorial.

## 3. Risk Factors for MASLD

### 3.1. Cardiovascular Disease

Cardiovascular disease is the most common cause of death among individuals with MASLD. Whether cardiovascular disease is an independent driver for MASLD remains unknown; however, current data suggest that cardiovascular risk is proportional to the degree of hepatic fibrosis. In a comprehensive meta-analysis involving 16 studies and over 34,000 participants, the existence of MASLD was linked to a 64% higher likelihood of both fatal and non-fatal cardiovascular events during a median follow-up of seven years. The risk escalated with the severity of the liver disease; however, it is noteworthy that traditional cardiovascular risk factors were not accounted for in the analysis. In a substantial analysis of European primary care databases, it was determined that, even after accounting for age, sex, smoking, and traditional metabolic risk factors (hypertension, Type 2 diabetes, high cholesterol, and statin use), there was no evident correlation between MASLD and the occurrence of myocardial infarction or stroke [9]. In an extensive meta-analysis performed in 2024, the relationship between MASLD and various cardiovascular conditions was evaluated. Drawing upon data from 32 studies encompassing over 5.6 million subjects, it was discovered that MASLD contributes to a heightened risk of conditions such as angina, coronary artery disease, the initial stages of coronary artery calcification, and the formation of calcified coronary plaques. However, the analysis did not establish a significant link between MASLD and advanced coronary artery calcification or myocardial infarction. These findings highlight MASLD’s role as an independent factor in the development of specific cardiovascular diseases, emphasizing the critical importance of early detection, prevention, and effective management of MASLD in reducing cardiovascular risk [10]. Furthermore, a comprehensive meta-analysis, which encompassed 36 studies totaling over 7 million subjects, examined the link between MASLD and cardiovascular disease (CVD); the incidence was examined. The findings indicated a substantial elevation in the risk for both non-fatal and fatal CVD events among MASLD patients. The hazard ratios were 1.57 for non-fatal CVD, 1.40 for fatal CVD, and 1.41 for a combination of the two. A pronounced increase in the risk of CVD was observed in MASLD patients with fibrosis, with a hazard ratio of 1.64. The analysis estimated an increased absolute risk of 29, 16, and 19 additional events per thousand individuals for combined, fatal, and non-fatal CVD events, respectively, associated with MASLD [11]. Finally, we included the last meta-analysis performed in 2023, which explored the relationship between MASLD and the thickness of the carotid artery intima–media (CIMT). This analysis encompassed 59 studies with a total of 42,299 participants, revealing that MASLD significantly correlates with an elevation of 0.1231 mm (20.6%) in the CIMT and an increased occurrence of atherosclerotic plaques in carotid arteries. This evidence highlights the potential of MASLD as a risk factor for carotid artery atherosclerosis. These findings underscore the necessity for further extensive prospective research to validate these correlations and to evaluate the effectiveness of current treatment strategies in mitigating these risks [12].

### 3.2. Dyslipidemia

Dyslipidemia is characterized by elevated triglyceride (TG) levels and low high-density lipoprotein cholesterol (HDL-C) and manifests in 69% of MASLD patients and 72% of those with MASH [13]. Non-alcoholic steatohepatitis has been linked to heightened levels of oxidized LDL-C, a well-established factor for atherosclerosis. Atherogenic dyslipidemia is prevalent in MASLD, and prolonged dyslipidemia may adversely impact lipid and lipoprotein synthesis in the liver by elevating the expression and activity of the sterol regulatory element binding protein-1c (SREBP-1c), a transcription factor. This, in turn, has detrimental effects on lipid and lipoprotein synthesis in the liver, leading to heightened levels of triglycerides (TG), low-density lipoprotein (LDL), and very low-density lipoprotein (VLDL), accompanied by reduced levels of high-density lipoprotein cholesterol (HDL-C). Interestingly, the effect of insulin resistance in patients with T2DM contributes to metabolic dyslipidemia, increasing the flux of free fatty acids, triglyceride production, and the production of very low-density lipoprotein (VLDL), along with oxidative stress and lipid peroxidation, all linked to MASLD development. Also, the apolipoprotein B/apolipoprotein AI (ApoB/AI) ratio has emerged as a valuable predictor for cardiovascular disease risk and is associated with MASLD prevalence [14]. Furthermore, a notable elevation in the risk of atherosclerotic plaque is found among individuals with liver fibrosis, indicating a potential association between liver disease and cardiovascular damage in dyslipidemic patients that goes beyond the insulin resistance hypothesis [13]. In essence, disruptions in the metabolism of fat lead to the accumulation of hepatic fat in MASLD, underscoring the intricate relationship between dyslipidemia, metabolic disorders, and liver health.

### 3.3. Type II Diabetes Mellitus

Among individuals diagnosed with MASLD, 51–60% coexist with obesity and 76% have type 2 diabetes mellitus (T2DM). Obesity resulting from a high-fat diet serves as the primary precursor initiating the pathways of lipotoxicity and glucotoxicity, both of which are mediated by insulin through insulin resistance (IR). Individuals with T2DM have cells that no longer respond to insulin, which normally functions as an anabolic hormone playing a role in maintaining fluid balance, facilitating ionic transport, storing triglycerides (TG) in adipose tissue, promoting the esterification and storage of fatty acids within lipid droplets, inhibiting the process of lipolysis, and suppressing the production of hepatic glucose while increasing the uptake of glucose peripherally. The severity of metabolic syndrome is correlated to the reduction in hepatic insulin clearance, and, furthermore, insulin has been linked with acting as an anti-inflammatory and proinflammatory agent. The term “insulin resistance” typically refers to the diminished responsiveness of cells, particularly in skeletal muscle, to normal levels of insulin. In critical situations, the body prioritizes glycolysis and the release of free fatty acids (FFAs) to meet peripheral demands, because a significant portion of glucose is directed to the brain. Consequently, hyperinsulinemia arises as beta cells attempt to compensate for insulin resistance by increasing insulin secretion. Excessive caloric intake damages insulin receptor signaling, leading to the impaired suppression of FFA release from adipose cells and compromised nitric oxide (NO) release. This creates a detrimental loop where insulin resistance and inflammation reinforce each other, hastening the progression of MASLD and other metabolic disorders [15]. Elevated insulin resistance was identified as the most substantial predictive factor for MASLD in individuals whether obese or lean [16], and, furthermore, serum insulin levels are strongly associated with ballooning and hepatic lobular inflammation [17].

### 3.4. Obesity

Obesity is a significant risk factor for MASLD and T2DM, and the development of these metabolic disorders results from an imbalance in energy input, consumption, and fat accumulation. Adipose tissue, acting as an endocrine organ, releases hormones and cytokines called adipokines. IR in MASLD is associated with the imbalance between proinsulin (adiponectin and leptin) and anti-insulin (e.g., TNFα) cytokines. Adiponectin, a specific adipokine, regulates fatty acid oxidation, inhibits free fatty acid accumulation, and maintains glucose homeostasis and hepatic insulin sensitivity. Low adiponectin levels contribute to fatty acid metabolism disturbances and chronic liver inflammation. Leptin, another adipokine, influences hepatic stellate cell activation, liver fibrosis, energy balance, and appetite suppression, and elevated leptin levels are observed in individuals with increased body fat and cardiometabolic disorders. A cross-sectional study revealed that MASLD patients exhibit lower adiponectin levels, higher serum leptin levels, and an increased leptin-to-adiponectin (L/A) ratio. Adiponectin and leptin independently predict MASLD onset, suggesting their potential as biomarkers for the condition. Additionally, researchers identified Gremlin 1, a novel adipokine, as a factor antagonizing insulin signaling. It is positively correlated with the body fat percentage and insulin resistance in T2DM and MASLD subjects [15]. Interestingly, in a 2024 cross-sectional analysis, the correlation between liver fibrosis, measured by the Enhanced Liver Fibrosis (ELF) test, and signs of myocardial injury and fibrosis, indicated by high-sensitivity cardiac troponin T and I levels, was examined in a group of 136 severely obese individuals. Initially, a positive correlation was observed between the ELF scores and elevated hs-cTnT and hs-cTnI levels. However, after adjusting for various confounding factors like age, gender, hypertension, and renal function, this correlation weakened significantly. The study therefore suggests that the apparent association between liver fibrosis and myocardial damage might be attributed to common underlying cardiovascular risk factors, rather than a direct causative link as initially hypothesized [18].

### 3.5. Iron Overload

Excess iron has been observed in individuals with MASLD, potentially playing a role in the development and advancement of the condition. Iron overload can impact lipid and glucose metabolism, potentially leading to insulin resistance, a pivotal factor in MASLD pathogenesis. Additionally, the surplus iron may contribute to the progression of MASLD by promoting oxidative stress and fibrogenesis within the liver. In a systematic review performed by Hernaez, a meta-analysis comprised 13 case–control studies from 1996 to 2010 and did not identify a substantial correlation between the presence of HFE mutations and MASLD among Caucasians. Specifically, the findings indicated that individuals with mutations in the HFE gene (C282Y/C282Y and C282Y/H63D) do not exhibit a higher risk of MASLD compared to controls. Even when controls were imputed from NHANES, the conclusions remained consistent, except for the presence of homozygosity. The discrepancy may be attributed to the overrepresentation of cases with C282Y homozygosity in case-only studies, which potentially masks a genuine association. In non-Caucasian populations, the analysis of three case–control studies suggested an excess of H63D mutants among MASLD cases compared to controls [19]. On the other hand, a meta-analysis of 43 studies with 5758 MASLD cases suggested an increased risk of MASLD associated with the C282Y polymorphism in Caucasians. The clinical and laboratory associations with MASLD included a higher median serum ferritin (SF) in probands with MASLD, indicating that MASLD contributes to hyperferritinemia but not iron overload. Elevated ALT and AST levels were more prevalent in probands with MASLD, but a significant association was not observed in logistic regression. Obesity prevalence did not differ significantly in probands with and without MASLD. T2DM was more prevalent in probands with MASLD, and its presence at the hemochromatosis diagnosis indicated the need for MASLD evaluation [20].

### 3.6. Galactosemia

Classical galactosemia represents a disorder within the monosaccharide metabolism that can induce fatty liver disease (FLD), particularly in symptomatic young infants. The intake of galactose from breastmilk or formula triggers widespread macrovesicular steatosis and cholestasis, leading ultimately to acute liver failure and mortality, unless galactose-free feeding is promptly initiated. In cases of severely ill newborns with cholestasis, galactosemia should be considered. A confirmation of the diagnosis involves measuring the galactose-1-phosphate-uridyltransferase (GALT) activity in erythrocytes or genetic analysis; normal GALT activity does not rule out galactosemia [21].

### 3.7. Alpha-1-Antitrypsin

Alpha-1-Antitrypsin (A1AT) deficiency (A1ATD) is a genetic condition primarily associated with lung disease, causing panacinar emphysema due to the impaired inhibition of the neutrophil elastase. Many mutations in the SERPINA1 gene, responsible for encoding A1AT, are linked to lung disease, and some of these mutations lead to liver disease. Liver disease in A1ATD is identified by specific pathological features, including PAS-positive, diastase-resistant inclusions. Despite various polymorphisms in the SERPINA1 gene, clinically significant liver disease is primarily associated with the Z mutant allele and, to a lesser extent, the S mutant allele [22]. Furthermore, the PiZZ homozygous variant represents the most severe manifestation of A1AT deficiency. Research conducted at the University of Iowa Hospitals and Clinics from 2005 to 2020 involved 1532 consecutive patients diagnosed with MASLD. The study revealed that the A1AT PiMZ variant significantly increased the likelihood of hepatic events in MASLD patients and independently predicted the development of such events. The findings highlight the importance of screening for the A1AT genotype in MASLD patients, and, for those identified with the PiMZ variant, considering aggressive interventions is crucial to mitigate their increased risk of developing decompensated cirrhosis [23].

### 3.8. Glycogen Storage Diseases

Glycogen Storage Diseases (GSDs) are a group of inherited metabolic disorders resulting from defects in enzymes involved in glycogen synthesis or degradation. Glycogen is a complex, branched polymer composed of glucose units. Following a meal, the glucose levels in the bloodstream rise, prompting the storage of excess glucose as glycogen in the cytoplasm. Among body tissues, the liver contains the highest proportion of glycogen by weight, approximately 10%, while muscles can store around 2% by weight. However, since the total mass of muscle tissue surpasses that of the liver, the overall glycogen mass in muscles is approximately double that of the liver. In times of need, the glycogen polymer can be enzymatically broken down into individual glucose molecules, which can then be used for energy production. Many enzymes and transporters play pivotal roles in these processes and are central to the development of Glycogen Storage Disorders (GSDs). Although an increasing number of GSDs are being discovered, most of them are exceptionally rare [24]. These diseases can be classified into those affecting the liver or those linked to neuromuscular issues. Glycogen Storage Disease Type IX (GSD-IX), a prevalent form (25% of cases), stems from a deficiency in an enzyme crucial for glycogen breakdown. This deficiency leads to elevated pyruvate levels, ultimately triggering liver lipogenesis and the synthesis of fatty acids and triglycerides. GSD-IX is both genetically and clinically diverse and is associated with a hepatic phosphorylase kinase (PhK) deficiency, with subtypes (GSD-IXa, GSD-IXb, GSD-IXc, GSD-IXd) identified by the affected subunit. These subtypes have different modes of transmission, and the common subtype GSD-IXa displays a wide range of symptoms. While most patients experience a benign course with mild childhood symptoms that improve over time, some may develop liver cirrhosis or liver adenomas and hepatocarcinoma in adulthood. In a research study case series, the challenges in diagnosing MASLD in children who were not obese but presented with hepatomegaly and elevated transaminases were highlighted. The study discusses the scarcity of guidelines for ruling out secondary causes of fatty liver, with glycogen storage disease type IX (GSD-IX) not mentioned in existing guidelines. GSD-IXa, identified through molecular study, is highlighted for its broad phenotypic spectrum and potential progression to cirrhosis. The study underscores the need for more appropriate and cost-effective guidelines for diagnosing secondary causes of MASLD, with GSD-IX suggested for inclusion due to its varied liver involvement spectrum. The use of a next-generation sequencing panel is proposed as a useful tool in managing these patients [25]. The research led by Rossi in 2020 demonstrated the efficacy of a high-fat dietary regimen in managing hepatic GSDs, with a focus on GSDIII and its impact on cardiomyopathy. Notably, the investigation revealed a decrease in blood CK levels and an enhancement in muscular strength among these patients. The results propose that a high-fat diet may be beneficial in diminishing cardiac glycogen accumulation and CK levels. The study also points out a differential impact of high-fat diets on liver enzymes based on age [26]. 

### 3.9. Viral Hepatitis

Traditionally, viral hepatitis has been a primary focus for both basic and clinical liver research, because it was previously the leading global indication of liver transplantation [9]. However, with the advent of effective therapies for hepatitis B and C, clinical attention has shifted. For hepatitis C, the focus is now on widespread treatment. In the case of hepatitis B, the emphasis is on lifelong antiviral therapy as needed, cancer surveillance for at-risk groups, and a transition towards global hepatitis B eradication through immunization and strategies for achieving a functional cure. According to a study by Wong in 2023, the chronic hepatitis B and fatty liver (CHB-FL) groups had a notably lower risk of mortality across various subgroups. This finding aligns with previous research on MASLD, which also indicated either no significant or reduced mortality in MASLD patients, particularly in the absence of advanced fibrosis. Additionally, MASLD-associated metabolic stress has the potential to activate innate and adaptive immunity, suppressing the hepatitis B virus and potentially delaying the disease progression in chronic hepatitis B patients through various mechanisms [27]. As hepatitis C can now be cured or patients can be given substantial treatment, other metabolic risk factors are now leading for liver disease. In accordance with the new nomenclature, hepatitis is now considered under the umbrella of cryptogenic steatotic liver disease (CSLD) and remains important to the pathogenesis of liver disease. Hepatic steatosis has been linked to an elevated risk of developing hepatocellular carcinoma and cirrhosis in individuals with chronic hepatitis B. However, it is worth noting that it also presents a greater opportunity for achieving a functional cure. This underscores the significance of detecting concurrent steatosis in chronic hepatitis B patients [28]. 

### 3.10. Wilson’s Disease

Wilson’s disease is a rare metabolic disorder characterized by the accumulation of copper in the liver and brain. In Japan, it affects 1 in every 40,000 individuals and is caused by mutations in the ATP7B gene, resulting in dysfunction of the copper-transporting enzyme P-type ATPase. This enzyme is crucial for copper transport into bile and incorporation into ceruloplasmin, a major copper-carrying protein in the blood. A reduced ceruloplasmin synthesis due to liver disease leads to decreased plasma levels, serving as an indicator of Wilson’s disease. The liver manifestations of Wilson’s disease can range from asymptomatic cases to various symptoms, including MASLD [29]. A study performed by Stättermayer focused on steatosis, an early liver pathology in Wilson’s disease (WD) resembling MASLD. A total of 98 Caucasian WD patients were examined, and it was found that 28 had moderate/severe steatosis, which was more prevalent in pediatric cases (50%) than in adults (20.8%); cirrhosis was present in 46.9% of patients. The study identified the PNPLA3 G allele and pediatric age as independent factors associated with moderate/severe steatosis. Interestingly, hepatic copper content did not significantly impact the severity of steatosis. The findings suggest that PNPLA3 genetic factors play a role in steatosis development in WD, emphasizing the need for further investigations into hepatic copper concentration and ATP7B mutations in this context [30]. Furthermore, in a study performed by Liggi, the patients with Wilson’s Disease (WD) and MASLD were compared, and the severity of steatosis was similar in both groups. No correlation was found between the level of steatosis and the metabolic factors studied. In patients with WD, the hepatic parenchymal copper concentration was significantly higher (753 ± 65.3 mcg/g dry weight) compared to in patients with MASLD (54.5 ± 16.9 mcg/g dry weight) (*p* < 0.05). Furthermore, within the WD group, the higher liver copper concentrations were associated with more severe steatosis compared to milder cases [31].

### 3.11. Cystic Fibrosis

Fatty liver disease is becoming more prominent among cystic fibrosis (CF) patients as awareness of MASLD grows. One study concluded approximately 30 to 40% of CF patients develop cystic fibrosis liver disease (CFLD), with half of them experiencing severe CFLD. Male gender is identified as the sole significantly associated risk factor, while a younger age at CFLD diagnosis correlates with a heightened risk of developing severe CFLD and is linked to a worse pulmonary function. CFLD poses challenges in definition, with the CFTR gene defect contributing to biliary damage, fibrosis, and cirrhosis, while CF-associated liver diseases encompass a spectrum from liver steatosis to steatohepatitis and idiopathic non-cirrhotic portal hypertension (INCPH) [32].

### 3.12. Leukocyte Telomere Length

A randomized control trial using data from the TONIC trial explored the relationship between leukocyte telomere length (LTL) and liver disease progression in children with MASLD. Unlike adults, in pediatric MASLD, no correlation was found between liver fibrosis and LTL. However, a longer LTL was associated with increased lobular inflammation both at baseline and after 96 weeks. This suggests that a longer LTL in children might indicate a higher risk of future complications from, MASH differing from the patterns observed in adult MASLD cases [33].

### 3.13. Smoking

**Figure 2:**
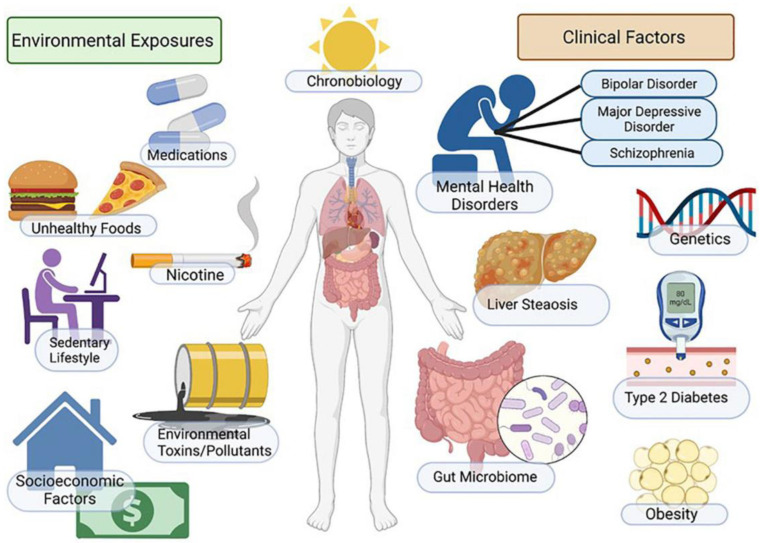
Risk Factors associated with MASLD [35].

Smoking is a preventable risk factor that is strongly associated with MASLD. Smoking is associated with many chronic diseases, such as cancer, insulin resistance, and T2DM, that are associated with MASLD. One study investigated the association between smoking and MASLD and the dependence of the association on smoking cessation duration and pack years. It indicated a dose-dependent negative association with the duration of smoking cessation and a positive association with pack years, suggesting that smoking cessation reduces MASLD incidence [34]. As shown in Figure 2, smoking is one of the many risk factors associated with MASLD [35].

## 4. Dietary Effects on Fatty Acid Metabolism

### 4.1. Gut Microbiome

The gut microbiome has been identified as an important factor in MASLD. Although more research is needed to determine the exact role that gut microbiome plays, dietary nutrients can lead to insulin resistance, metabolic endotoxemia, low-grade inflammation, the development of obesity, metabolic syndrome, and MASLD [36,37,38,39,40]. The proposed mechanism of the effect of the gut microbiome on MASLD includes increased energy harvest, increased intestinal permeability, altered metabolites, and increased microbial endotoxins [41,42].

Altered gut microbiomes increase caloric retention due to the increase in energy production pathways seen in patients with MASLD [41].

An imbalance between gut microbiome composition increases membrane permeability, allowing bacteria to enter portal circulation and reach the liver. This exposure to damaging substances has been proposed as a cause of the development of MASLD. Studies have associated increased intestinal permeability with the degree of MASH [41,43,44].

Altered metabolites have been shown to contribute to MASLD pathogenesis in animals and are an area of interest for further research. The role of short-chain fatty acids (SCFA) in MASLD remains controversial. Some studies show an increased insulin sensitivity and the reduction of fat storage via G protein signaling, AMPK, and regulating NF-κβ, TNF-α, and IL-1β. Other studies show excessive SCFAs may inhibit AMPK and increase the accumulation of hepatic free fatty acids, as well as increasing proinflammatory T-cells (Th1, Th17) under specific conditions [41,45,46].

Bile acids aid in the connection between the gut microbiome and the liver. Primary bile acids activate the Farnesoid X receptor (FXR), which induces the FGF19 to activate mTORC1 via MAPK, which enhances the glucose uptake into adipocytes. FXR also limits hepatic lipid accumulation and increases FA beta-oxidation by inhibiting SREBP-1c and inducing PPARα. Secondary bile acids activate Takeda G-protein-coupled Receptor 5 (TGR5), which increases insulin synthesis while decreasing appetite by releasing GLP-1. FXR and TGR5 combined suppress bile acid regulation by inhibiting cholesterol 7α-hydroxylase (CYP7A1). A healthy gut microbiome maintains the intestinal barrier and provides anti-inflammatory actions [41,47,48].

Choline is a metabolite that has been implicated in the pathogenesis of MASLD. Choline is a cell membrane phospholipid that comes from dietary intake and endogenous synthesis. The metabolite is needed for VLDL production, and a deficiency could lead to triglyceride accumulation and the progression of MASLD. Choline metabolism by the gut microbiome produces phosphatidylcholine and trimethylamine (TMA), which are further metabolized by trimethylamine-N-oxidase (TMAO). Proteobacteria, firmicutes, and actinobacteria all contribute to this metabolism and further decrease choline bioavailability. Although the mechanism of the TMAO contribution to MASLD is unclear, patients with MASLD have a higher concentration of serum TMAO compared to healthy controls [49]. Elevated TMA and TMAO levels are associated with an elevated ratio of firmicutes to Bacteroidetes, which is common in the gut microbiome of MASLD patients [41,50].

Endogenous ethanol is a microbial metabolite that is markedly increased by carbohydrate-rich diets. MASLD patients have more Escherichia, Gammaproteobacteria, and Prevotella than healthy controls. All of these are alcohol-producing gut bacteria and lead to higher levels of serum ethanol in patients with MASH who do not consume alcohol. Ethanol metabolism stimulates lipogenesis and inhibits fatty acid oxidation. Ethanol increases gut permeability and inflammation and disrupts the apical junctional complexes in the colon epithelium. Ethanol metabolism produces ROS and exacerbates the oxidative stress by inducing CYP2E1, mitochondrial ROS production, and mitochondrial DNA damage. Acetaldehyde is produced when ethanol is converted by CYP2E1, which can lead to hepatotoxicity when the pathway is saturated and acetaldehyde accumulates [41,51,52]. 

Increased microbial endotoxins may play a role in MASLD. The increase in Gram-negative bacteria (proteobacteria, enterobacteria, and escherichia), accompanied with increased permeability, elevates the serum endotoxin lipopolysaccharides that are released from the cell walls of these bacteria. Inflammation and plasma endotoxin levels are markedly increased in MASLD patients [41,53].

### 4.2. Gut–Liver Axis

The gut–liver axis is disrupted in MASLD patients. An unhealthy liver cannot regulate the gut microbiome, and an unregulated gut microbiome leads to worse liver health. Altering the gut microbiome has the potential to alleviate this cycle. This can be done by direct manipulation of the gut microbiota by removing harmful strains, introducing beneficial strains, or altering microbial metabolites. The removal of harmful stains can be done using antibiotics, antimycotics, or bacteriophages. The introduction of beneficial strains can be done with probiotics or by isolating beneficial strains from healthy individuals. There can be the community restoration of the entire microbiota by utilizing fecal microbiota transplant. Targeting metabolic pathways that induce or repress microbial metabolites has the potential to be beneficial to MASLD patients. The microbiome also has the potential to be used for diagnosis of MASLD by looking at microbial metabolites such as succinate, phenylacetic acid, and 3-(4-hydroxyphenyl) lactate. Diet and medication are the main disruptors responsible for the changes in our microbiome leading to MASLD [54].

### 4.3. Western Diet/Fatty Diet

The western diet consists of large portions, which are high in saturated fats, trans fats, and added sugars, while being low in fruits and vegetables [55]. The accumulation of monounsaturated fats due to a western diet exacerbates oxidative damage and hepatic steatosis in early MASLD, and possibly contributes to the progression of MASLD [56,57]. Fructose emerges as a significant contributor to its development and progression, going beyond calorie overconsumption and weight gain. Found in sweetened beverages and processed foods, fructose boosts lipogenesis by providing substrates for fatty acid synthesis through aldolase B and ketohexokinase actions. It also activates transcription factors such as sterol regulatory element-binding protein 1c (SREBP1c). A meta-analysis, encompassing over 2000 individuals, suggests that excess energy from sugar-sweetened beverages, primarily containing fructose, contributes to an augmentation of liver fat [8]. Interestingly, the literature highlights the hepatoprotective potential of coffee in individuals with MASLD, elucidating its mechanisms encompassing antioxidative, anti-inflammatory, and antifibrotic effects, and chemopreventive properties. These findings contribute to understanding the positive impact of coffee consumption on individuals with chronic liver disease [58].

### 4.4. Mediterranean Diet

The Mediterranean diet is rich in whole grains, fish, monounsaturated fats, antioxidants, polyphenols, vitamins, and fiber, while maintaining a low intake of saturated fats and animal proteins [39,59]. This diet has been shown to decrease cardiovascular disease, T2DM, and metabolic syndrome. Although research is limited on this diet, the potential to improve liver health makes its use an attractive option for MASLD patients [39,59]. Adherence to the Mediterranean diet, characterized by components like olive oil rich in monounsaturated fatty acids and/or omega-3 fatty acids, demonstrates benefits beyond weight loss. Even in the absence of weight reduction, this dietary approach not only diminishes liver steatosis but also enhances insulin sensitivity among individuals with MASLD and insulin resistance. Furthermore, studies indicate that an imbalance between dietary omega-3 and omega-6 fatty acids, with a deficiency in the former and an increase in the latter, can contribute to the development of MASLD in rodents [58]. 

A PREDIMED clinical trial examined the impact of three diets on liver steatosis: a Mediterranean Diet (MedDiet) supplemented with extra-virgin olive oil, a MedDiet with nuts, and a low-fat control diet. Involving 100 individuals at a high risk of cardiovascular disease, the study found that the MedDiet + extra-virgin olive oil group had a notably lower prevalence of hepatic steatosis after three years compared to the other groups. This suggests that an unrestricted MedDiet supplemented with extra-virgin olive oil could be beneficial in reducing liver fat in older individuals with a high cardiovascular risk [60].

A systematic review and meta-analysis evaluated the impact of ginger supplementation on MASLD, which is closely associated with obesity and insulin resistance. An analysis of 18 studies, including both in vivo experiments and clinical trials, revealed that ginger significantly improved liver and serum markers in MASLD. Notably, there were reductions in liver cholesterol, triglycerides, and malondialdehyde, and increases in catalase and superoxide dismutase. Serum markers like ALT, AST, TG, LDL, and total cholesterol also showed improvements, along with increases in HDL and decreases in fasting blood sugar. These benefits are attributed to ginger’s insulin-sensitizing, antioxidant, and antidyslipidemic effects, and its role in reducing hepatic fat content and ROS generation. The findings suggest the potential therapeutic application of ginger in FLD [61].

Silymarin, derived from milk thistle seeds originating in the Mediterranean basin, is known for its hepatoprotective properties, particularly in cases of liver diseases such as cirrhosis and fatty liver disease. Clinical studies using the Eurosil 85^®^ formulation have highlighted its efficacy in reducing liver-related deaths and improving liver function. Notably, silymarin’s antioxidant activity plays a crucial role in scavenging free radicals that cause lipid peroxidation and cellular damage. Its benefits are most pronounced when treatment begins early in liver disease progression, leveraging the liver’s regenerative potential and countering oxidative stress-induced cytotoxicity [62]. 

### 4.5. Intermittent Fasting

A comprehensive meta-analysis performed in 2023 investigated the impact of various intermittent fasting (IF) strategies, such as the 5:2 diet, 16/8 time-restricted feeding, and alternate-day fasting, on cardiometabolic and hepatic indicators in individuals with MASLD. An analysis of 7 randomized controlled trials, drawn from an initial pool of 12,343 articles, revealed that these IF protocols, administered over 2 to 3 months, markedly lowered liver steatosis scores and metrics associated with obesity (including body mass index, overall body weight, and waist circumference). Additionally, significant reductions were noted in ALT, triglycerides, total cholesterol, HbA1c, and HOMA-IR levels. Conversely, parameters such as fasting glucose, insulin, AST, HDL-C, LDL-C, and liver fibrosis scores did not show significant changes. These findings suggest that IF may offer certain benefits for cardiometabolic and liver health in MASLD patients [63].

## 5. Genetic Pathways Related to MASLD

### 5.1. Oxidative Stress

Oxidative stress plays a key role in the development of metabolic dysfunction-associated steatohepatitis (MASH) from simple steatosis [64]. The excess lipids in hepatocytes cause mitochondria to increase β-oxidation to eliminate these free fatty acids, promoting the production of reactive oxygen species (ROS) in the respiratory chain. In the same manner, the excess lipids in the liver with MASH further develop the ROS, suppressing the electron transport chain function, insulin sensitivity, and lipid metabolism, while inducing inflammatory responses and cellular damage [65]. The oxidative stress saturates antioxidant mechanisms, triggering lipid peroxidation by polyunsaturated fatty acids. This leads to the increased formation of ROS and reactive nitrogen species (RNS) that diffuse into the extracellular space contributing to tissue damage [53,66].

The oxidative stress activates the Kupffer cells (KCs), resulting in the release of proinflammatory cytokines like TNFa and IL-1b. These cytokines play a role in the inflammatory response, worsening damage to hepatocytes. The KCs recognize the damage-related molecular patterns (DAMPs) that are released by injured cells, recruiting monocytes and neutrophils to foster inflammation collectively. One such DAMP is the high mobility group box-1 protein (HMGB1), which binds to toll-like receptor 4 (TLR4) on KCs [67]. This binding activates the NF-κB signaling pathway, leading to the substantial release of proinflammatory factors and escalating inflammatory reactions. Additionally, DAMPs can activate TLR9 on KCs, amplifying TLR9-dependent ROS production and the release of inflammatory mediators, further enhancing the inflammatory process and damage to the liver [68].

The ROS induces damage to the mitochondria by altering the electron transport chain, increasing membrane permeability and impairing the ability of the mitochondria to reduce ROS levels [69]. The ROS activates TNF-α, phosphorylating c-Jun-N-terminal kinase (JNK) and producing more ROS. The accumulation of ROS induces the mitochondrial apoptosis pathway by opening the mitochondrial membrane permeability transition pore (MPTP) [70,71]. As a result, the electron transport chain is uncoupled, damaging the mitochondrial membrane. Cytochrome C released into the cell combines with apoptosis activator 1, activating caspase-9 and caspase-3 leading to apoptosis [72].

Interestingly, ginger supplementation significantly improves liver and serum markers in non-alcoholic fatty liver disease (MASLD). The improvements include reductions in liver cholesterol, triglycerides, and malondialdehyde, as well as increased levels of catalase and superoxide dismutase. These positive effects are attributed to ginger’s insulin-sensitizing, antioxidant, and antidyslipidemic effects, and its role in reducing hepatic fat content and ROS generation, making it a potential therapeutic option for patients with MASLD [61]. Other studies have also investigated the possible role of food treating the accumulation of ROS. A recent trial looking at the efficacy of curcumin with piperine supplementation in reducing oxidative stress in patients with MASLD was assessed in a double-blind, placebo-controlled trial of 55 participants. The study measured the serum pro-oxidant and antioxidant balance (PAB) before and after the intervention. The results indicated that the supplementation did not significantly alter the serum PAB levels compared to the placebo group, suggesting that the provided dosage might be insufficient to decrease PAB values in these patients effectively [73].

### 5.2. Genetic Mutations

Mutations in CIDEB and HSD17B13 offer protective roles in patients with MASLD. The CIDEB gene promotes the growth and storage of lipids in the liver [74,75]. Loss of function mutations to this gene play a protective role against MASLD [76]. 

The exact role of the 17-Beta Hydroxysteroid Dehydrogenase 13 (HSD17B13) gene is unknown. The enzymatic activity of HSD17B13 under conditions of obesity, infection, and alcoholism contributes to ALD and MASH. Loss of function mutations in this gene prevent liver injury, inflammation, fibrosis, cirrhosis, and HCC [77,78].

Genetic mutations, such as PNPLA3, TM6SF2, GCKR, MBOAT7, APOC3, and CYP2E1, can be detrimental in patients with MASLD.

PNPLA3: The rs738409 C to G variant of PNPLA3 (Patatin-like phospholipase domain-containing protein 3) encodes the I148M allele and is said to be the strongest genetic risk factor for MASLD. PNPLA3 is a multifunctional enzyme involved in the hydrolysis and accumulation of triacylglycerol in the liver. The I148M allele is linked to the severity progression and mortality of MASLD [2,79,80].

Transmembrane 6 superfamily member 2 (TM6SF2) E167K variant (loss of function) is associated with an altered VLDL synthesis and secretion allowing triglycerides to accumulate in the liver while also lowering serum cholesterol and lipids [79]. The accumulation of lipids in the liver secondary to E167K variants has been associated with damage to the liver [80]. Due to the decrease in circulating triglycerides, this variant is also associated with a decrease in cardiovascular risk [79]. Thus, the TM6SF2 E167K variant has been identified as an independent risk factor for MASLD and increased hepatic triglyceride content but has not been associated with the severity of the disease [81].

Glucokinase regulator (GCKR) is an allosteric inhibitor of glucokinase (GCK) and is important in regulating glucose homeostasis. Glucokinase is the first step in glycolysis and phosphorylates glucose, keeping it inside the hepatocyte. Loss of function variant GCKR—rs1260326 C to T SNP, which encodes for the P446L protein, allows for the GCK to go unregulated by the GCKR and continually generate precursors (malonyl CoA) for fatty acid synthesis through glycolysis. This variant is associated with steatosis in the liver and an increased MASLD risk. The rs780094 C to T, intron variant is linked to high triglyceride levels and MASLD severity [79].

Membrane Bound O-Acetyltransferase Domain Containing 7 (MBOAT7) expression is decreased in patients with obesity and is linked to liver damage and alterations in fat metabolism that could lead to MASLD [82].

The APOC3 rs2854116 polymorphism has been associated with increased triglycerides and may contribute to MASLD risk in the Asian population [83].

The overexpression of cytochrome CYP2E1 has long been associated with MASLD by promoting oxidative stress, inflammation, protein modification, and insulin resistance [84,85,86,87]. The recent literature has shown that CYP2E1 is necessary for the progression of the disease but does not trigger MASLD on its own. CYP2E1 requires elevated hepatic lipids from lipogenic diets or increased hepatic lipogenesis to have pathophysiological effects [88]. Uncontrolled diabetes can induce CYP2E1 leading to MASLD [89,90].

### 5.3. Epigenetics

Epigenetics plays a key role in the development and progression of MASLD. TGF-β1, Collagen 1A1, and platelet-derived growth factor (PDGF) are all genes involved in fibrosis. The hypomethylation of these genes in the more advanced stages of MASLD upregulates their expression, increasing the risk of fibrogenesis [91,92].

Insulin-like growth factor-binding protein (IGFBP)-2: The hypermethylation of Insulin-like growth factor-binding protein (IGFBP)-2 is seen in patients with MASLD and MASH. The hypermethylation of IGFBP2 has also been associated with the development of T2DM and suggests a relationship between these two diseases [93].

ENPP1 121 Gln and IRS-1 972 Arg polymorphisms affect the insulin receptor activity, leading to a decrease in insulin signaling. These variants suggest a causal role in the progression of MASLD [94].

Peroxisome Proliferator-Activated receptor gamma coactivator (PGC)-1α regulates the fatty acid oxidation, mitochondrial biogenesis, and various other aspects of energy metabolism. The methylation of the promoter sequence in PGC-1α is seen in patients with MASLD. The decreased expression of PGC-1α is associated with mitochondrial defects and insulin resistance [79].

SIRT-1 is involved in regulating hepatic lipid metabolism, oxidative stress, and inflammation. Reduced levels of SIRT-1 are found in MASLD patients, but the reduction of SIRT-1 alone is not enough to induce the disease, indicating the role of epigenetics. miR-34 targets SIRT1 and is overexpressed in MASLD patients. miR-34 reduces mitochondrial oxidation, leading to the buildup of lipids in the liver [79,95].

### 5.4. MicroRNA Posttranscriptional Regulation

MicroRNA (miRNA) plays an important role in the posttranscriptional regulation of key genes involved in MASLD [96].

miR-122: The gene expressions of Acetyl-CoA carboxylase 2 (ACC2), ChREBP, PPARγ, PPARα, and SREBP are regulated by miR-122. The expression of these genes is essential for lipid metabolism. In MASLD patients, hepatic levels of miRNA are reduced, while serum levels are elevated [79].

miR-34a: The overexpression of miR-34a leads to decreased fatty acid oxidation, along with increased inflammation, ROS, and apoptosis, and the progression of MASLD [97].

miR-21: miR-21 may be implicated in lipid accumulation, steatosis, inflammation, and fibrosis of MASLD [98].

miR-155: It has been suggested that miR-155 participates in hepatic injury, steatosis, and fibrosis involved in MASLD [99].

miR-29: The effect of miR-29a is controversial. miR-29a has been shown to have a detrimental effect on liver fibrosis. While other studies show miR-19a has a protective effect against MASLD [100], miR-29a has been shown to inhibit GSK3b, leading to a decrease in steatosis, steatohepatitis, and steatofibrosis. miR-19 has therefore been shown to have protective effects against MASLD [101].

miR-192: miR-192 has been shown to progress MASLD by activating proinflammatory macrophages [101].

miR-375: The effect of miR-375 is also controversial. miR-375 has been shown to be upregulated in steatosis and MASLD. miR-375 has been shown to have protective effects by regulating adipokines and MASLD progression [102].

## 6. The Future with Organoids

Organoids mimic both the structure and function of their original tissue [103,104]. The evolution of liver organoids has transitioned from early single-cell epithelial types to more complex structures that both incorporate parenchymal cells (hepatocytes or cholangiocytes) and support non-parenchymal cells such as Kupffer cells and HSCs [105].

Organoids are 3D miniaturized versions of tissues and organs. The cells are incubated in 3D culture systems, where they aggregate, self-organize, and differentiate into 3D masses resembling the organ tissue morphology of their corresponding origins. These cells have stem cell potential and are very similar to parental cells, maintaining unique characteristics by conserving parent gene expression and mutation characteristics [106].

Organoids are far superior to their 2D counterparts because they maintain the morphology, cell-to-cell interactions, metabolism, and behavior of their parent tissues/organs. Organoids can better represent tissues and organs due to the 3D physicochemical microenvironment that the 3D culture system provides [104,106].

Organoids are cultivated from pluripotent stem cells (PSC), induced PSCs (iPSC), embryonic stem cells (ESCs), adult stem cells (ASCs), and tumor cells using a variety of techniques [107]. The submerged culture technique involves embedding cells in an extracellular matrix and placing the mixture in a culture dish to form a dome. The dome is then submerged in a medium that provides nutrients and the required factors for tissue growth [106,108,109,110].

**Figure 3:**
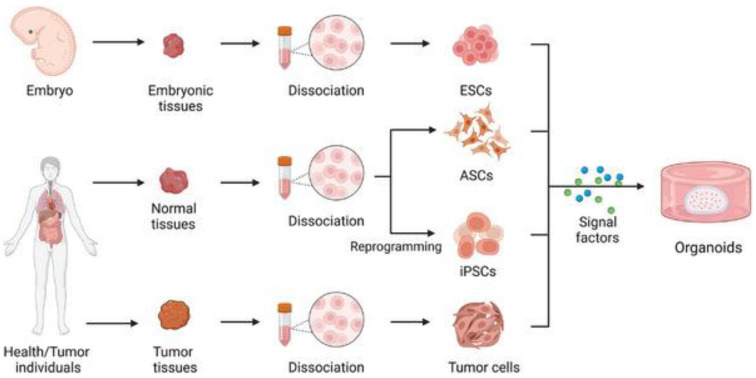
Strategies for the formation of organoids in vitro. Organoids can be cultivated from several types of cellular origins, such as embryonic stem cells, stem cells derived from adults, cells reprogrammed to an embryonic-like pluripotent state known as induced pluripotent stem cells, and cells from tumors. Tissue samples obtained through surgical resection or biopsy from healthy or diseased individuals are separated into individual cells. These cells are then grown into organoids in a controlled environment supplemented with a range of signaling molecules [106].

Air–liquid interface (ALI) technology uses mechanically separated tissue fragments homogeneously embedded in collagen gel that is laid in an inner culture dish with a porous membrane. A medium is added to the outer culture dish that allows nutrients and required growth substances to diffuse into the inner dish. This is shown further in Figure 3 [106,111,112].

The Organ-on-a-chip is a microfluidic cell culture device that allows for control of the biochemical and biophysical environment for tissue growth. This device simulates cellular conditions, microenvironment conditions, inter-tissue interactions, and multiorgan interactions. The Organ-on-a-chip technique allows for much better control of the tissue growth environment. Organoid-on-a-chip combines organ-on-a-chip and organoid technologies allowing cells to spontaneously self-organize into 3D structures [106,113,114].

Organoids have a variety of uses from drug development to personalized medicine. They can be used for drug research, screening, and toxicity assays. Organoids allow for disease modeling and studying organ-specific genetic diseases, infectious diseases, metabolic diseases, and cancers [115,116,117,118,119,120]. Adipose organoids have been used to study metabolism and obesity associated with T2DM and MASLD [121]. Steatosis has been studied using iPSC-derived liver organoids with hepatocytes, stellate, and Kupffer-like cells by increasing lipid content, inflammation, and fibrosis. Tumor organoids can be constructed from a biopsy, and drug sensitivity predictions for individual patients can be analyzed, allowing for personalized medicine. Organoids can be used for regenerative medicine to restore the normal structure and function by repairing or replacing damaged cells, tissues, or organs. Organoids cultured in vitro pose an alternative source for organ donations that can combat the various challenges that transplants pose, such as graft rejection or the unavailability of needed organs. Organoid transplantation in animals has proven successful at restoring lost organ functions [106,122].

Recent advancements have been made in utilizing three-dimensional liver organoids as a tool for investigating the pathophysiology of MASLD and MASH, along with potential treatments. Various techniques are employed in creating liver organoids, comparing induced pluripotent stem cells with primary cell lines and human with murine cells, and methods have been explored to induce lipid droplet accumulation and fibrosis for MASLD modeling; the overarching goal of recent studies is to replicate the intricate liver microenvironment where MASLD evolves and progresses to MASH [123,124].

De novo lipogenesis, the main contributor to fatty liver, involves potential therapeutic targets like SREBP-1c, ChREBP, ACC, and FAS, with various drug candidates in trials [105,125]. Monocellular epithelial liver organoids, specifically hepatocyte organoids and cholangiocyte organoids, have been proposed as effective models for drug evaluation, demonstrated in the screening of MASLD drugs [105,126,127]. Oxidative stress, resulting from excessive ROS production, has been implicated in MASLD, with ongoing investigations into antioxidants such as vitamin E. Monocellular epithelial liver organoids have been suggested as a model for assessing antioxidant effectiveness. Cell death mechanisms, including apoptosis, necroptosis, pyroptosis, and ferroptosis, contribute to MASH progression. Promising anti-cell death agents like Emricasan, Selonsertib, and Rapamycin have been explored in preclinical studies [105,128,129]. Monocellular epithelial liver organoids are viewed as suitable models for evaluating these agents across various cell death pathways, providing a comprehensive platform for drug development and enhancing our understanding of the MASLD and MASH mechanisms [105,130].

Although organoids have progressed dramatically over the past years there are limitations to the technology. Few laboratories are able to culture organoids. The organoids that are developed lack cellular maturity and typically resemble fetal tissues. The extracellular matrix in which the organoids are grown is inconsistent and has the potential to introduce viral or xenogenic contaminants [106,131].

## 7. Therapy

### 7.1. Lifestyle

It is widely accepted that the current guidelines for the first line treatment of MASLD is lifestyle intervention, including diet modulation and physical activity [132]. Increasing physical activity has been shown to have positive effects on MASLD, independent of weight loss [133]. Research has shown physical activity consisting of high or medium aerobic intensity while using resistance training improves the status of elevated liver enzymes, total cholesterol, triglycerides, and intrahepatic fat [134]. Participating in aerobic exercise not only increases the activity and expression of antioxidant enzymes but also contributes to the mitigation of oxidative damage induced by dichlorodiphenyltrichloroethane (DDT). This reduction is mainly attributed to the improved availability of oxygen during aerobic activities [58]. Nonetheless, combining diet and exercise is a more effective treatment than either on their own [135]. However, overall, the most reliable and consistent indicator of MASLD resolution is the extent of weight loss. Substantial weight loss not only results in a decreased supply of fatty acids to the liver but also diminishes inflammation in the adipose tissue, lowers proinflammatory cytokine secretion, and enhances insulin sensitivity. These improvements subsequently contribute to positive changes in liver histology [58]. Healthy diet principles, such as the Mediterranean diet and a high protein diet, are recommended. It has been proven that if individuals with MASLD lose 5–7% of their body weight, they can reverse steatosis. Furthermore, if they lose up to 10% of their body weight, they can improve any hepatic fibrosis [134].

### 7.2. Bariatric Surgery

Bariatric surgery has been suggested as an effective treatment option for MASLD as weight loss is a cornerstone for treatment. Bariatric surgery is considered safe, enhances conditions such as steatosis and inflammation, improves fibrosis scores, and lowers the risk of mortality associated with cardiovascular disease and hepatocellular carcinoma linked to MASLD [136]. Bariatric surgery also offers substantial weight loss and hepatic improvements independent of weight loss via improvements in lipid metabolism, T2DM, and insulin resistance. Bariatric surgery is not without risk, and long-term studies that demonstrate the efficacy for MASLD are lacking [41,137].

### 7.3. Transplant Surgery

Liver transplantation (LT) is the sole lifesaving option for MASH -related end-stage liver disease and unresectable HCC, making MASLD the second leading cause for LT in the USA (21.5% of transplants in 2018) and meaning that LT is experiencing exponential growth in Europe (1.2% in 2002 to 8.4% in 2016). The patients who have had liver transplants as a result of MASH often present with HCC, a higher age, and an increased BMI. The mechanisms contributing to the higher prevalence of HCC in MASH include chronic inflammation, genetic polymorphisms, increased iron absorption, gut dysbiosis, lipid storage, insulin resistance, and elevated insulin-like growth factor levels. Additionally, a notable proportion of HCC in MASLD/MASH arises in a non-cirrhotic liver, as demonstrated in studies from Italy, Germany, and Japan [138]. The 5-year survival rate for individuals with MASLD-related hepatocellular carcinoma is reported to be 85% [9].

**Figure 4:**
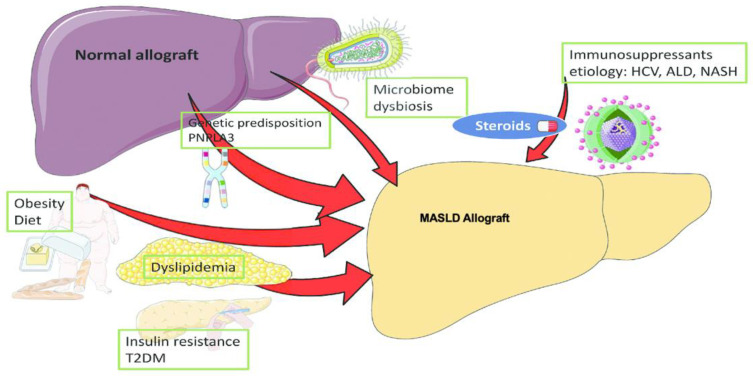
Pathogenic factors involved in pre-transplant MASLD are suspected to also contribute tothe post-transplant development of MASLD [139].

Individuals following liver transplantation face a risk of developing recurrent or de novo MASLD, and, although the precise mechanisms underlying this development remain unclear, there is speculation that similar processes may be at play as the development of primary MASLD. The risk factors in developing post-transplant MASLD are demonstrated in Figure 4, and furthermore include are an elevated pre-transplant BMI, substantial weight gain after the transplant, pre-existing metabolic syndrome in cases of MASH cirrhosis prior to transplantation, and an increased probability of developing a metabolic syndrome post-transplant due to factors like immunosuppressant use and the characteristics of the donor graft [139].

### 7.4. Pharmacology

Currently, there is no approved drug therapy by either the European Medicines Agency or the United States Food and Drug Administration (FDA) for MASLD, highlighting the urgent requirement for the creation of effective treatments for this widespread health issue [140]. However, a broad category available for the treatment management of MASLD includes antioxidants like vitamins C and E, insulin-sensitizing agents to improve the indirect causes of MASLD like DM, and hepatoprotective agents such as thioglitazones, Ursodeoxycholic acid, statins, pentoxifylline, and Orlistat [58]. The drugs that are currently under development are targeting the gut microbiome to improve MASLD [141]. Many pharmacological targets exist and are marketed in the drug development pipeline for future approval to treat MASLD: THRβ, lipogenesis inhibitors, ACC inhibitors, bile acid metabolism modulators, fibrogenesis inhibitors, glucose metabolism modulators, mesenchymal stromal cells, fraudulent fatty acids like bempedoic acid and gemcabene, tesamorelin for growth hormone modulation, berberine ursodeoxycholate for AMPK activation, miricorilant as a glucocorticoid receptor modulator, nitazoxanide with AMPK activation and HSC inhibition, pirfenidone with antifibrotic and anti-inflammatory properties, and other agents such a gut microbiome. These diverse strategies showcase the evolving landscape of potential treatments for different stages of MASLD [140].

### 7.5. Vitamin E

One comprehensive meta-analysis performed in 2023 examined the influence of vitamin E supplementation on serum aminotransferase levels (AST and ALT) in adults with MASLD. The findings indicate that vitamin E dosages ranging from 400 to 800 IU effectively reduced the serum levels of ALT and AST, with a more pronounced effect on ALT. These outcomes were consistent across different ethnic groups and were not affected by concurrent dietary or exercise modifications. Notably, the positive impact of vitamin E was evident in both short-term (up to 24 weeks) and longer-term (48 weeks and beyond) studies. A specific focus on pediatric MASLD revealed a more significant improvement in the ALT levels among children and adolescents. However, concerns regarding the accuracy of the results from a biased adolescent study necessitated its exclusion, leading to more balanced ALT and AST level changes in the revised analysis. Safety concerns around high-dose vitamin E supplementation, particularly regarding a cancer risk, are notable. Despite challenges like diverse study designs, the varied forms of vitamin E supplementation used, and small participant numbers, this review offers an in-depth evaluation of vitamin E’s role in managing liver enzyme levels in MASLD, backed by the latest clinical trials and detailed subgroup analyses [142].

### 7.6. Glucose Metabolism Modulators

There is therapeutic potential of various pharmacological agents targeting nuclear receptors and metabolic pathways. The peroxisome proliferator-activated receptors (PPARs), including PPARα, PPARγ, and PPARδ, play crucial roles in regulating glucose homeostasis, lipid metabolism, and inflammatory responses. Selective PPAR agonists, such as fenofibrate and saroglitazar, demonstrate varied efficacy in resolving the biochemical and histological aspects of MASLD [143]. Additionally, inhibitors of mitochondrial pyruvate carrier (MPC), such as azemiglitazone and PXL065, and incretin mimetics like semaglutide and tirzepatide exhibit promising effects on liver function and histology. Sodium/glucose cotransporter 2 (SGLT2) inhibitors, like dapagliflozin and empagliflozin, demonstrate improvements in liver dysfunction, steatosis, and fibrosis, emphasizing their potential as therapeutic agents for MASLD [144]. Lastly, glucagon-like peptide 1 (GLP1) receptor agonists and α-glucosidase inhibitors have positive effects for MASLD treatment [140].

### 7.7. GLP-1 Receptor Agonist Drugs

Recent analytical findings indicate that for MASLD, particularly when concurrent with type 2 diabetes, the use of SGLT-2 inhibitors and GLP-1 receptor agonists is beneficial. Among these, semaglutide, liraglutide, and dapagliflozin have been identified as particularly effective, with semaglutide demonstrating a superior therapeutic efficacy. The effect of these medications varies across different health indicators, suggesting a need for tailoring treatments based on individual patient conditions. Despite their efficacy in MASLD management, the current evidence does not comprehensively address their effects on liver morphology, underlining the necessity for direct comparative clinical trials to solidify these findings [145].

### 7.8. THRβ

The thyroid hormone receptor beta (THRβ) is a crucial nuclear receptor in the human liver, activated by compounds like triiodothyronine. It plays a key role in gene transcription, enhancing processes such as free fatty acid uptake, oxidation, and mitochondrial biogenesis [146]. THRβ activation also influences transcriptional processes related to bile acid synthesis and lipid clearance, reducing proatherogenic lipoproteins. Selective THRβ agonists like resmetirom, VK2809, TERN501, ASC41, and MGL-3745 are under development for MASLD treatment, showing promising results in clinical trials. Fixed-dose combinations like ASC43 and ASC45 are also being explored for their therapeutic efficacy [147].

### 7.9. Lipogenesis Inhibitors

Key enzymes involved in de novo lipogenesis, including ACC, FASN, SCD1, and DGAT, are crucial targets for treating MASLD [148]. However, the insights from animal studies indicate that inhibiting TAG synthesis may worsen hepatic inflammation and fibrosis. Additionally, it may impact intestinal barrier function, leading to diarrhea and steatorrhea. These findings present challenges in developing lipogenesis inhibitors, potentially limiting their clinical application to the early stages of liver steatosis not associated with inflammation and fibrogenesis [149].

### 7.10. ACC, FASN, SCDI, and DGAT Inhibitors, and PUFA

Numerous investigational compounds are being explored for the treatment of MASLD, targeting key enzymes and pathways involved in lipid metabolism. Selective ACC inhibitors currently under development for combination therapy demonstrate substantial improvements in the reduction of MASLD activity, liver steatosis, lobular inflammation, hepatocellular ballooning, and liver biochemistry [150] Clesacostat (2–50 mg/d) exhibited effectiveness in reducing liver steatosis and is being developed in conjunction with the DGAT2 inhibitor ervogastat to address the rise in serum TAG associated with ACC inhibitors. Ongoing phase 2 studies seek to establish the optimal doses for MASH, both with and without liver fibrosis (NCT04399538, NCT04321031) [151]. FASN inhibitors like ASC40 have shown promising results in inhibiting de novo lipogenesis and reducing liver steatosis, with ongoing studies like FASCINATE-2 [152]. The SCD1 inhibitor Aramchol demonstrated effects on lipogenesis, fibrogenesis, and serum cholesterol levels in the ARREST trial, with the ARMOR RCT in progress [153]. DGAT inhibitors, including Ervogastat and ION224, are undergoing trials, and ω-3 polyunsaturated fatty acids (PUFAs) like ALA, EPA, and DHA have shown efficacy in reducing liver steatosis and inflammation. Compounds like epeleuton and icosabutate are being investigated for their potential in MASLD treatment. These findings offer diverse therapeutic avenues for addressing various aspects of MASLD pathology. Ongoing research aims to further establish the safety and efficacy of these compounds in clinical settings [154,155,156].

### 7.11. Bile Acid Metabolism Modulators

The farnesoid X receptor (FXR), a bile acid receptor predominantly found in the liver and ileum, regulates various metabolic processes, including cholesterol and TAG metabolism. FXR activation represses lipogenic pathways and is a promising target for MASLD therapy [157] FXR-activating drug candidates include obeticholic acid (OCA), EDP-305, INT-767, and non-steroidal compounds like MET409, tropifexor, cilofexor, vonafexor, and TERN-101 [140]. OCA, the first-in-class FXR agonist, is approved for non-cirrhotic primary biliary cholangitis and is in phase 3 trials for MASH. Several newer compounds aim to optimize FXR agonists, while dual FXR/TGR5 agonists like INT-767 and BAR502 are being explored [158,159]. Fibroblast growth factors (FGF) 19 and 21, downstream messengers of FXR, are also being investigated, with aldafermin showing promise in MASH. Other FGF analogues like efruxifermin and BIO89-100 are in development for MASLD treatment [160]. Overall, the FXR and FGF pathways present multifaceted targets for addressing various aspects of MASLD pathology.

### 7.12. Fibrogenesis Inhibitors

Galectins, such as galectin-1, -3, and -9, are implicated in liver disease, playing diverse roles in fibrogenesis, immunity, inflammation, and tumorigenesis. Galectin-1 promotes fibrogenesis but can also benefit liver regeneration. Galectin-3 regulates hepatitis, fibrosis, cirrhosis, and hepatocellular carcinoma progression, with potential protective effects against certain conditions [161]. Drug candidates targeting galectins, like belapectin and GM-CT-01, are being explored for advanced MASH with fibrosis or cirrhosis. Toll-like receptor 4 (TLR4) antagonists, LOXL2 inhibitors, and autotaxin (ATX) inhibitors are being investigated for their roles in liver fibrosis. PPAR agonists, including seladelpar and saroglitazar, are being studied for their impact on glucose metabolism and MASH, showing promise in clinical trials [162,163,164].

## 8. Conclusions

This comprehensive research on non-alcoholic fatty liver disease (NAFLD), rebranded as metabolic dysfunction-associated steatotic liver disease (MASLD), illuminates its multifaceted nature. The disease’s progression, influenced by factors like oxidative stress, metabolic risk factors, genetic predispositions, and lifestyle elements, underscores the complexity of its management. Innovative approaches like dietary modifications, surgical and pharmacological interventions, and the emerging use of organoids present promising avenues for treatment. The evolving understanding of MASLD’s pathology, especially its interplay with cardiovascular diseases and metabolic syndromes, highlights the need for personalized and multi-disciplinary treatment strategies. This research reinforces the critical importance of early intervention and comprehensive management in addressing this growing global health concern.

## Figures and Tables

**Table 1:** Overview of MASLD.

Section	Subsection	Focus Area
1. Introduction	-	Overview of MASLD
2. Pathophysiology	-	Mechanisms behind MASLD
3. Risk Factors	3.1. Cardiovascular Disease	Link between MASLD and cardiovascular health
	3.2. Dyslipidemia	Impact of abnormal lipid levels on MASLD
	3.3. Type II Diabetes Mellitus	Connection between diabetes and MASLD
	3.4. Obesity	Role of obesity in MASLD development
	3.5. Iron Overload	Influence of iron on MASLD
	3.6. Galactosemia	Association of galactosemia with MASLD
	3.7. Alpha-1-Antitrypsin	A1AT deficiency’s effect on MASLD
	3.8. Glycogen Storage Diseases	GSDs and their contribution to MASLD
	3.9. Viral Hepatitis	Hepatitis and MASLD relationship
	3.10. Wilson’s Disease	Wilson’s Disease and its association with MASLD
	3.11. Cystic Fibrosis	CFLD’s relationship with MASLD
	3.12. Leukocyte Telomere Length	Leukocyte TL in relation to MASLD development
	3.13. Smoking	Smoking as a risk factor for MASLD
4. Dietary Effect on Fatty Acid Metabolism	4.1. Gut Microbiome	Gut microbiome’s impact on MASLD
	4.2. Gut–Liver Axis	Interaction between gut and liver in MASLD
	4.3. Western Diet/Fatty Diet	Influence of diet on MASLD
	4.4. Mediterranean Diet	Benefits of this diet for MASLD
	4.5. Intermittent Fasting	IF’s effect on MASLD
5. Genetic Pathways Related to MASLD	5.1. Oxidative Stress	Role of oxidative stress in MASLD
	5.2. Genetic Mutations	Key genetic factors in MASLD
	5.3. Epigenetics	Epigenetic influences on MASLD
	5.4. MicroRNA Posttranscriptional Regulation	miRNA’s role in MASLD
6. The Future with Organoids	-	Application of organoids in MASLD research
7. Therapy	7.1. Lifestyle	Lifestyle interventions for MASLD
	7.2. Bariatric Surgery	Surgical options for MASLD treatment
	7.3. Transplant Surgery	Liver transplantation in MASLD management
	7.4. Pharmacology	Drug treatments for MASLD
	7.5. Vitamin E	Role of vitamin E in MASLD treatment
	7.6. Glucose Metabolism Modulators	Medications affecting glucose metabolism
	7.7. GLP-1 Receptor Agonist	GLP-1’s therapeutic potential in MASLD
	7.8. THRβ	THRβ agonists in MASLD treatment
	7.9. Lipogenesis Inhibitors	Targeting lipogenesis in MASLD
	7.10. ACC, FASN, SCDI, DGAT Inhibitors and PUFA	Role of inhibitors in MASLD treatment
	7.11. Bile Acid Metabolism Modulators	Bile acid’s impact on MASLD
	7.12. Fibrogenesis Inhibitors	Inhibiting fibrogenesis in MASLD treatment
8. Conclusions	-	Summary of findings and future directions
